# Different uptake patterns of ^68^Ga-FAPI in aseptic loosening and periprosthetic joint infection of hip arthroplasty: A case series and literature review

**DOI:** 10.3389/fmed.2022.1014463

**Published:** 2022-11-24

**Authors:** Yiqun Wang, Ruimin Wang, Lei Geng, Qingxiao Li, Erpeng Qi, Yuanyuan Shi, Yanmei Wang, Qingyuan Zheng, Guoqiang Zhang, Jiying Chen, Jiahe Tian

**Affiliations:** ^1^Department of Nuclear Medicine, The First Medical Centre, Chinese PLA General Hospital, Beijing, China; ^2^Beijing Key Laboratory of Sports Injuries, Department of Sports Medicine, Institute of Sports Medicine of Peking University, Peking University Third Hospital, Beijing, China; ^3^Department of Orthopedics Surgery, The First Medical Center, Chinese PLA General Hospital, Beijing, China; ^4^Department of Orthopedics Surgery, The Fourth Medical Centre, Chinese PLA General Hospital, Beijing, China; ^5^Department of Interventional Ultrasound, The First Medical Centre, Chinese PLA General Hospital, Beijing, China; ^6^General Electric (GE) Healthcare China, Shanghai, China

**Keywords:** ^68^Ga-FAPI, PET/CT, hip, arthroplasty, image analysis, case report

## Abstract

**Background:**

The diagnosis of a periprosthetic joint infection (PJI) is always a difficult point in research on the surgery of joints. The current diagnostic criteria include a comprehensive analysis of multiple tests; however, there are no effective visual examinations yet that can differentiate between aseptic loosening and the PJI.

**Case presentation:**

This case report describes four patients with symptomatic total hip arthroplasty (THA), two cases of loosening and two cases of infection. Although the four cases were correctly diagnosed by the tissue culture, preoperative tests and pathological examination could not effectively distinguish an infection from a non-infection. Based on a preclinical study and theoretical feasibility, gallium-68 (^68^Ga)-labeled fibroblast activation protein inhibitor positron emission tomography/computed tomography (^68^Ga-FAPI PET/CT) was performed. Through ^68^Ga-FAPI PET/CT scanning, not only were the causes diagnosed correctly but the lesions were also located.

**Conclusion:**

When the lesion is located between the bone and the prosthesis, ^68^Ga-FAPI PET/CT could differentiate aseptic loosening from periprosthetic joint infection (PJI). ^68^Ga-FAPI PET/CT has clear advantages over routine examinations and has a prospective application in detecting PJI.

## Introduction

Periprosthetic joint infection (PJI) is one of the most devastating complications in total joint arthroplasty. The incidence of PJI varies from 1 to 15% and places a heavy burden on patients and their families and the health system (1, 2).

Prompt and correct diagnosis is the premise of any effective treatment. Neither the Musculoskeletal Infection Society (MSIS) criteria proposed in 2011 (3) nor the International Consensus Group (International Consensus Meeting (ICM)) criteria developed in 2018 (4) are effective in the diagnosis of PJI, and the diagnosis does not rely on a single test. However, it should be noted that nuclear medicine-related examinations are rarely included in any of the diagnostic criteria.

Gallium-68 (^68^Ga)-labeled fibroblast activation protein inhibitor ^68^Ga-FAPI) is one of the radionuclides that is currently of great interest (5, 6).

Fibroblast activation protein (FAP) is mainly secreted by activated fibroblasts and plays an important role in inflammation, infection, and immunity (7). A literature review reported that a fibrous membrane is produced by the loosening of prosthesis (8) and that all typical signs of infection, such as granulomas and fibrosis, have fibroblast expression. Therefore, theoretically, ^68^Ga-FAPI has the potential for aseptic loosening and PJI imaging. In our previous animal studies (9, 10), ^68^Ga-FAPI, compared with 18F-fluorodeoxyglucose (^18^F-FDG), has clear advantages, including no uptake of muscles and the intestine, showing a wide range of lesions and a higher sensitivity to disease detection. Although the performance characteristics of the loosening model and the infection model are totally different, the maximum standardized uptake value (SUVmax) alone cannot effectively distinguish between the models of loosening and infection, so speculating the e performance characteristics of ^68^Ga-FAPI in clinical cases is crucial.

In this study, four typical cases, including aseptic loosening of the acetabular cup, PJI of the acetabular cup, aseptic loosening of the femoral stem, and PJI of the femoral stem and acetabular cup, are presented. Using the ^68^Ga-FAPI PET/CT [gallium-68 (^68^Ga)-labeled fibroblast activation protein inhibitor positron emission tomography/computed tomography] not only confirmed the final diagnosis but also helped determine the location and extent of the lesions.

## Case series

This case series report was approved by the Institutional Review Board of the Chinese People's Liberation Army General Hospital and followed CARE (Case Report) guidelines (Supplementary Figure). Cases were identified in our department between January and February 2021. Written, informed consent forms and permission to use patient data for scientific purposes were obtained at the time of patient's examination. The final diagnosis was based on the ICM criteria in 2018.

### Case 1: Aseptic loosening of the acetabular cup

**Figure 1:**
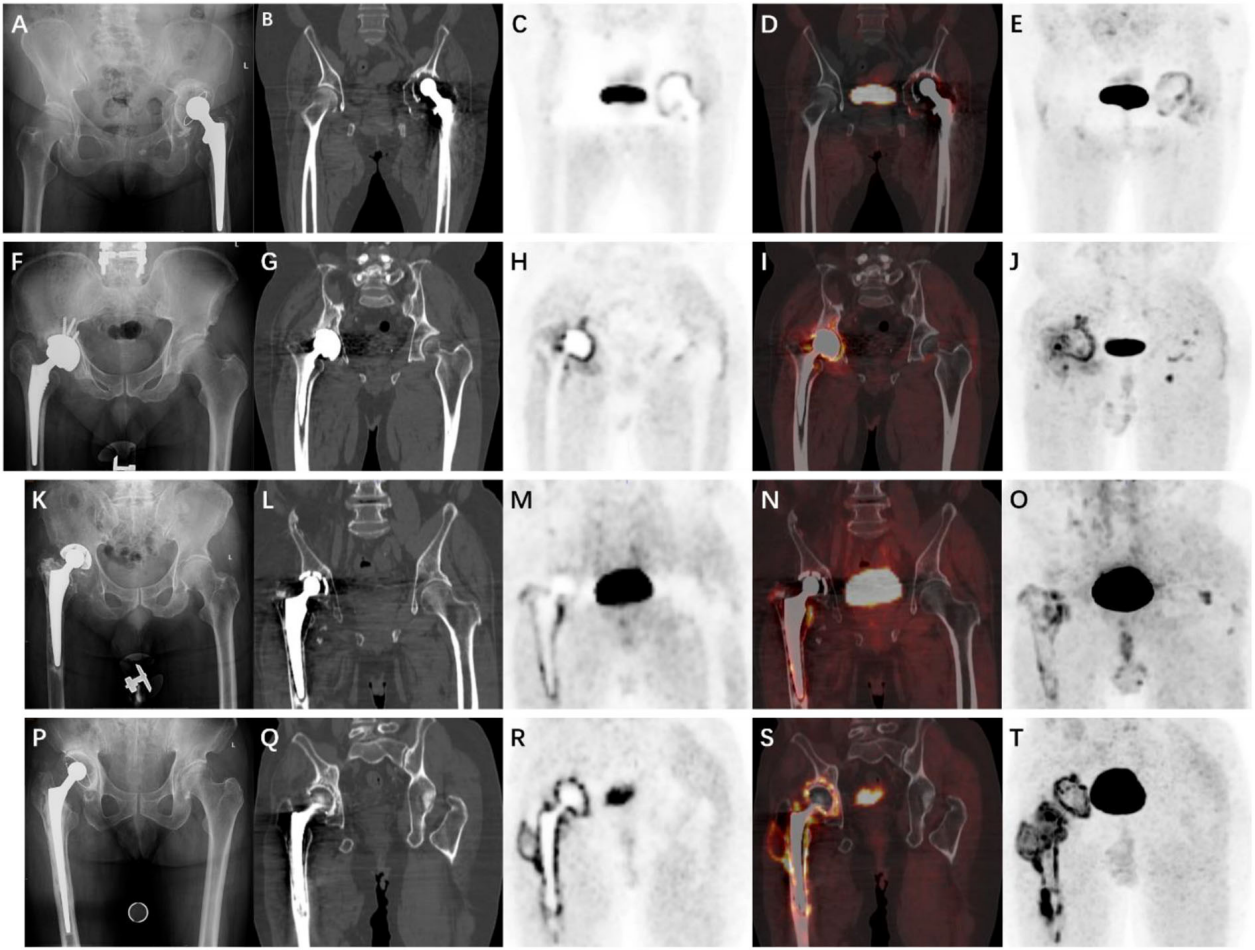
X-ray and ^68^Ga-FAPI PET/CT [gallium-68 (^68^Ga)-labeled fibroblast activation protein inhibitor positron emission tomography/computed tomography] images of each patient. **(A–E)** Case 1. **(F–J)** Case 2. **(K–O)** Case 3. **(P–T)** Case 4. First column: X-rays. Second column: Computed tomography (CT) images. Third column: Positron emission tomography (PET) images. Fourth column: Fusion images. Fifth column: Maximum intensity projection (MIP).

A 60-year-old female patient complained of left hip pain for ~2 years. She had cemented total hip arthroplasty (THA) for 9 years. Her serological and synovial tests were normal (Table 1). X-ray and ^68^Ga-FAPI PET/CT were performed (Figure 1). The x-ray results showed osteolysis around the acetabular cup, and PET/CT scan images showed moderate uptake around the periacetabular cup and the proximal femoral stem. This case was diagnosed as an aseptic loosening, and the acetabular cup was easily removed during the operation. Pathology and tissue culture were all negative.

**Table 1:** Results of examinations.

**Test**	**WBC**	**CRP**	**D-dimer**	**ESR**	**IL-6**	**Synovial**	**Synovial**	**Pathology**	**SUVmax**
	**(10^9^/L)**	**(mg/dl)**	**(μg/ml)**	**(mm/h)**	**(pg/ml)**	**WBC**	**PMN (%)**		
Case 1	5.88	0.05	3.6	16	2.55	0.112	55	–	6.6
Case 2	9	0.515	4	25	1.5	/	/	+	13.31
Case 3	5.65	0.05	0.5	13	8.6	/	/	+	8.55
Case 4	5.63	1.967	0.9	28	13.3	7	72	+	20.08

Normal range of examinations: WBC < 10, CRP < 1, D-dimer < 0.86, ESR < 30, IL-6 < 1.5, Synovial WBC < 3, Synovial PMN% < 80.

WBC, white blood cells; CRP, C-reactive protein; ESR, erythrocyte sedimentation rate; IL-6, interleukin 6; PMN%: polymorphonuclear leukocytes; SUVmax, maximum standard unit value.

### Case 2: PJI of the acetabular cup

A 60-year-old male patient presented to our orthopedic center due to failure of THA. He had a cementless THA 9 months ago. However, because of the wrong implant size, the patient began to complain of pain in the left hip immediately after surgery. There were no visible abnormalities on the preoperative examinations. The x-ray results showed a slightly smaller prosthesis, and ^68^Ga-FAPI PET/CT showed high-intensity uptake along the entire acetabular cup, which was totally different from the first case. This uptake pattern was believed to be indicative of PJI. Given the preoperative diagnosis and patient's history, one-stage reconstruction was chosen. However, the pathology revealed more than 20 neutrophils in 10 high-power fields on the frozen section slides, and the tissue culture revealed a mixed infection of *Acinetobacter baumannii* and *Staphylococcus epidermidis*. Therefore, the final diagnosis of this patient was PJI.

### Case 3: Aseptic loosening of the femoral stem

A 76-year-old male patient complained of left hip pain for 3 years. He had a THA 20 years ago, and serological results were normal, except for interleukin 6 (IL-6) (Table 1). No clear finding was discovered on x-ray. ^68^Ga-FAPI PET/CT showed moderate uptake along the entire femoral stem (Figure 1). This case was diagnosed as aseptic loosening; meanwhile, the pathology results were positive, and the tissue culture was negative.

### Case 4: PJI of the femoral stem and the acetabular cup

**Figure 2:**
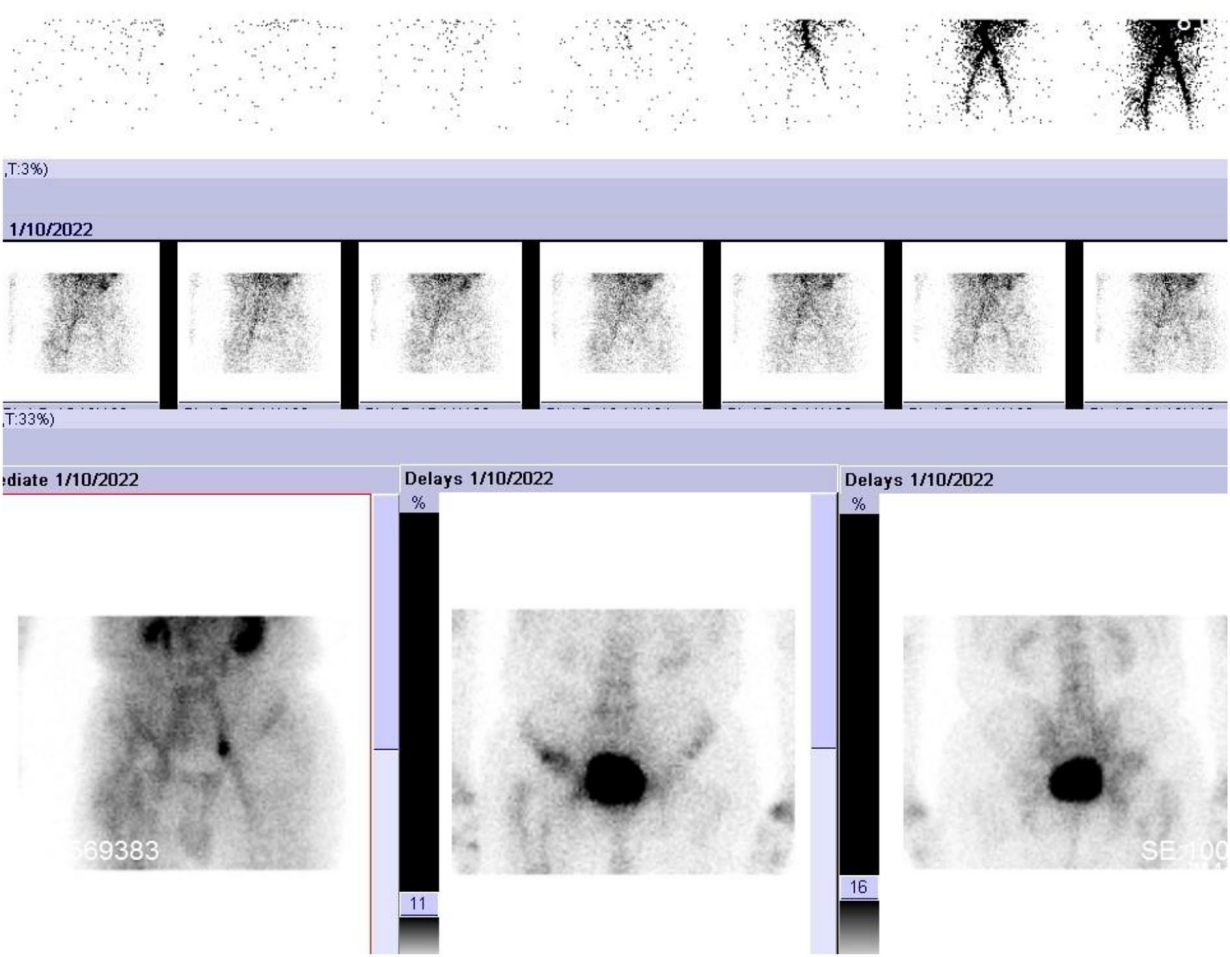
Triple-phase bone scanning of Case 4.

A 68-year-old male patient was admitted to our orthopedic center due to persistent right hip pain for ~3 years. He underwent primary THA 13 years ago, and after 3 years, the patient underwent revision surgery for aseptic loosening. The patient was diagnosed with an iliac fossa abscess 1 year ago, and a blood culture revealed the presence of *Klebsiella pneumoniae*. Recently, the patient's hip joint symptoms worsened, and serological results showed elevated C-reactive protein (CRP) and IL-6 and synovial white blood cell (WBC) counts were also elevated (Table 1). X-ray, triple-phase bone scintigraphy, and ^68^Ga-FAPI PET/CT were also performed. The x-ray results were inconclusive, and PET/CT showed intense uptake around the entire acetabular cup and the femoral stem (Figure 1). For triple-phase bone scintigraphy, although the uptake in the late phase was not strong, the three phases were all positive (Figure 2), which was indicative of infection. Intraoperative pathology showed 5–10 neutrophils in 10 high-power fields, and a two-stage reconstruction was chosen. The pathogen was identified as *Klebsiella pneumoniae* by intraoperative tissue culture.

## Discussion

In 2011, the Musculoskeletal Infection Society (MSIS) proposed the diagnostic criteria for PJI (3). Since then, the American Academy of Orthopedic Surgeons (AAOS), the Infectious Diseases Society of America (IDSA), the European Bone and Joint Society (EBJIS), and the International Consensus Meeting on Periprosthetic Joint Infection have also developed their criteria for PJI. Currently, the diagnosis of PJI is becoming increasingly mature and standard, and it no longer depends on a single test, but many researchers are also looking forward to finding a more accurate and even perfect examination. What are the advantages and disadvantages of commonly used tests in clinical practice?

Erythrocyte sedimentation rate (ESR) and C-reactive protein (CRP) are the most commonly used serological tests, which are economical and quick to obtain results. However, their specificity is not ideal, as underlying inflammatory joint diseases can also lead to elevated outcomes. In addition, their thresholds are different in different reports. Another point to note is that serological tests depend on the host's response to pathogens. For low-toxic, latent bacterial infection, serological tests may increase the probability of false negatives, and for patients with other diseases, such as pneumonia, erysipelas, cellulitis, and tumors, serological tests are likely to increase the possibility of false positives.

The synovial fluid analysis mainly consisted of WBC count, leukocyte esterase (LE)%, and polymorphonuclear leukocyte (PMN)%. WBC count and PMN% are considered to be highly sensitive and specific for PJI diagnosis; however, no clear thresholds are given in either MSIS or in IDSA diagnostic criteria, and the threshold varies greatly among different reports (11). The reasons attributed to the difference in the threshold values are thought to be as follows: (1) joint hematoma or postoperative inflammatory reaction will lead to a change in the threshold; (2) many studies exclude inflammatory joint disease, leading to unreliable results; and (3) metal-on-metal hip arthroplasty may lead to false positives. LE was originally used to detect urinary tract infections (UTIs) and was proposed for the diagnosis of PJI in ~2010. However, in the LE study, many cases were excluded due to excessive blood and debris in the articular fluid or vague results, which in turn also led to unreliable results (11).

The advantage of histopathology is that it is not affected by preoperative antibiotics. Using intraoperative freezing can help obtain the results in the operating room and guide treatment. However, its accuracy depends on the experience of pathologists, and some bacteria may not cause sustained neutrophil infiltration. In addition, although the most commonly used criterion is a mean of five or more polymorphonuclear leukocytes (PMN%) seen in five high-power fields, the standards reported are not uniform (12). In addition, some scholars proposed the classification according to the periprosthetic interface membrane, including wear particle-induced type, infectious type, combined type, and indeterminate type. This classification is not as widely used as the previous one, but it suggested that the presence of infection was not merely neutrophil infiltration and that neutrophil infiltration is not necessarily an infection (8).

Tissue culture is a useful test, but obtaining only a single specimen reduces the sensitivity of the test and makes it difficult to interpret contamination. The current standard is to take five specimens during the operation, and two positive cultures of the same organism are defined as PJI. If only one is positive, then other tests need to be combined (3, 4). However, when the infection is located between the bone and the prosthesis or in the biofilm, this method becomes inadequate. Therefore, some scholars use sonication of the removed prostheses to diagnose infection. This method uses colony-forming units per milliliter (CFU/ml) for diagnosis. Although most of the results calculated from the receiver operating characteristic (ROC) curve were satisfactory, the threshold of CFU/ml reported varied from 1 to 200 (13). Moreover, the process of sonication requires many steps, and the standards of different institutions are not uniform, which increase the uncertainty and unreliability of the results. In addition, sonication also requires tissue culture, and the choice of culture medium and culture days can also influence the results.

Through the analysis of the commonly used tests, we found that, regardless of the serological test, synovial analysis, or tissue culture, they all have an insurmountable defect, namely, the inability to locate infections, and in clinical practice, the determination of the infection boundary is usually dependent on experience. At present, there are many reports describing approximately 1.5-stage or partial two-stage reconstruction (14, 15), in which the infected part is exchanged and the normal part is not treated. Although these methods could reduce the trauma of the operation and the burden of medical treatment, there is no suitable test that can be used as the basis for these treatments.

From triple-phase bone scintigraphy, which has been used for the longest time, to the radiolabelled leukocytes, known as the “gold standard” imaging technique for PJI, and to ^18^F-FDG, which is the most widely used radionuclide in the clinic, scholars have never stopped their attempts to make an imaging diagnosis of PJI, and the results of the same tests are inconsistent, and each examination has limitations that cannot be overcome. For example, the limitations of single-photon emission computerized tomography (SPECT/CT) include high radiation dose, long examination time, and unsatisfactory resolution, while in our previous report, the imaging of the blood vessels, the intestines, and the muscles of ^18^F-FDG has a certain influence on diagnosis, and ^18^F-FDG cannot reflect the situation of soft tissue remodeling, as some cases had nonspecific uptake for years after surgery. PET/CT itself has certain advantages, but a more specific radionuclide is needed. Based on our previous animal experiments and theoretical feasibility, ^68^Ga-FAPI was selected in this study (9, 10, 16).

Regarding the classification proposed by Reinartz et al. (17), based on the ^18^F-FDG uptake pattern, our previous report noted a deficiency in this classification, namely, the absence of an infection between the bone and the prosthesis. In this report, ^68^Ga-FAPI showed a completely different uptake pattern in aseptic loosening and PJI. For aseptic loosening, the uptake pattern is mainly characterized by low-intensity linear uptake between the bone and the prosthesis, while the uptake pattern of PJI is characterized by high-intensity diffuse uptake. Through the literature review, this is the first report that demonstrated the different manifestations of loosening and infection on PET/CT of the acetabular cup and the femoral stem.

Meanwhile, this report has several limitations. First, only four cases are presented here, and a large number of cohort studies are warranted to verify this diagnostic criterion based on the uptake pattern. Second, all the patients had a gap of at least 6 months between THA and PET/CT scans, and whether the early period after THA influences ^68^Ga-FAPI results is also worth researching.

## Conclusions

In this study, we reported four symptomatic patients with THA. Compared with routine tests, ^68^Ga-FAPI PET/CT not only diagnosed correctly but also determined the location and extent of the lesion. This advantage is of great implication for 1.5-stage or partial two-stage reconstruction. Although related studies are warranted, ^68^Ga-FAPI has shown an important application value in PJI diagnosis.

## Data availability statement

The original contributions presented in the study are included in the article/Supplementary material, further inquiries can be directed to the corresponding author/s.

## Ethics statement

The studies involving human participants were reviewed and approved by institutional review board of Chinese People's Liberation Army General Hospital. The patients/participants provided their written informed consent to participate in this study. Written informed consent was obtained from the individual(s) for the publication of any potentially identifiable images or data included in this article.

## Author contributions

YiW, RW, and LG wrote the original draft. YS, YaW, and QZ collected and interpreted the data. QL and EQ designed the project. GZ, JC, and JT reviewed and edited the draft. All authors contributed to the article and approved the submitted version.

## Conflict of interest

The authors declare that the research was conducted in the absence of any commercial or financial relationships that could be construed as a potential conflict of interest.

## Publisher's note

All claims expressed in this article are solely those of the authors and do not necessarily represent those of their affiliated organizations, or those of the publisher, the editors and the reviewers. Any product that may be evaluated in this article, or claim that may be made by its manufacturer, is not guaranteed or endorsed by the publisher.
