# Supplementary figures and images for: Different uptake patterns of 68Ga-FAPI in aseptic loosening and periprosthetic joint infection of hip arthroplasty: A case series and literature review
Source: Front Med (Lausanne). 2022 Nov 24;9:1014463. doi: 10.3389/fmed.2022.1014463 (PMC9729767; doi:10.3389/fmed.2022.1014463)

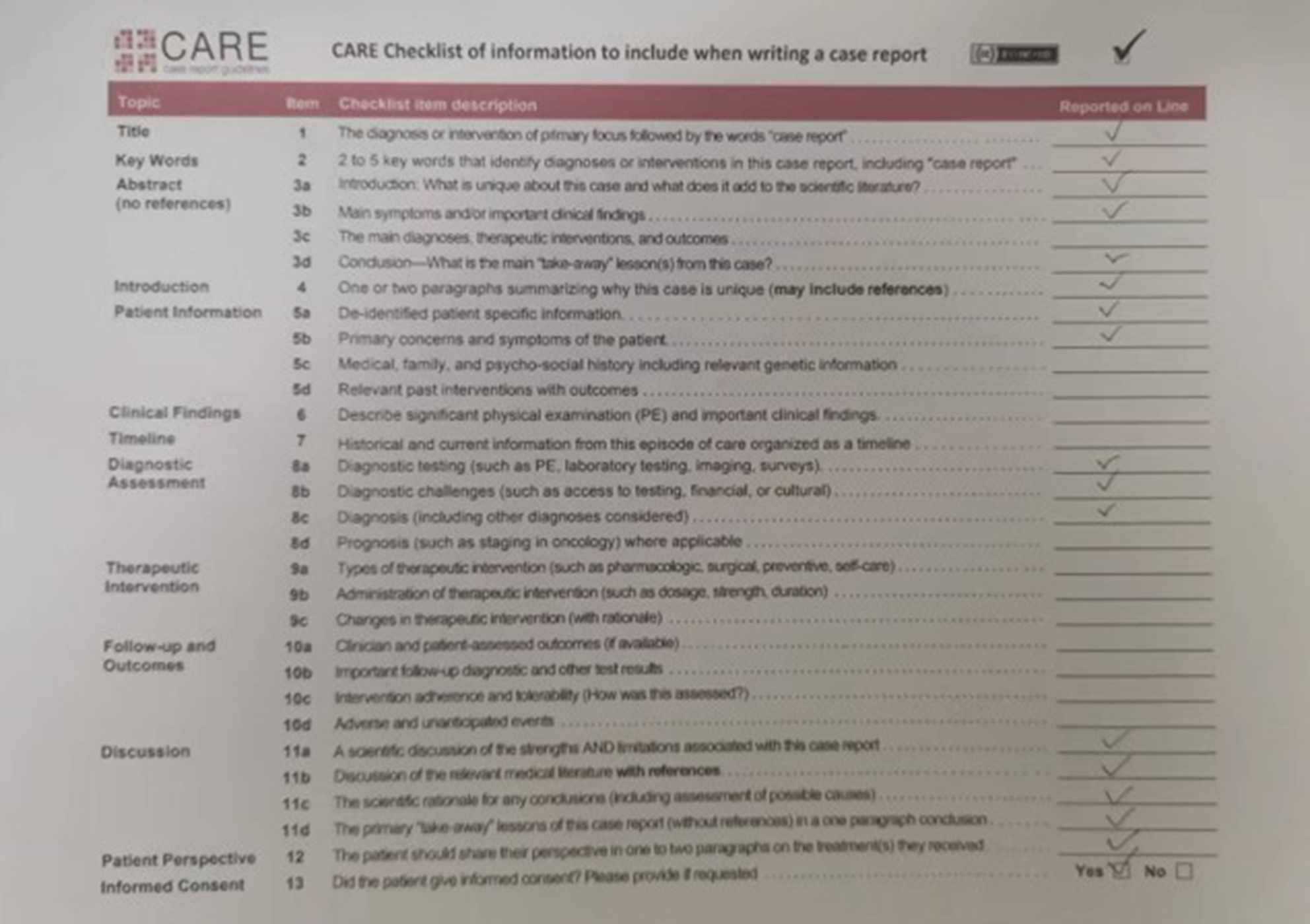

Supplement: Supplementary file 1 [file Image_1.JPEG]
